# Work-Related Musculoskeletal Disorders and Associated Factors Among Bankers in Ethiopia, 2018

**DOI:** 10.1155/2020/8735169

**Published:** 2020-09-08

**Authors:** Alemu Kasaw Kibret, Berihu Fisseha Gebremeskel, Kebede Embaye Gezae, Gebrerufael Solomon Tsegay

**Affiliations:** ^1^Department of Physiotherapy, School of Medicine, College of Health Sciences and Ayder Comprehensive Specialized Hospital, Mekelle University, Mekelle, Tigray, Ethiopia; ^2^Department of Biostatistics, School of Public Health, College of Health Sciences, Mekelle University, Mekelle, Tigray, Ethiopia

## Abstract

**Background:**

Work-related musculoskeletal disorders (WMSDs) are an important public health problem in working environments. WMSDs are the major causes of disability and cause individual suffering and financial burdens to the individual, families, industry or employer, healthcare system, and society at large. This study aims to assess the prevalence and associated factors of work-related musculoskeletal disorders among bankers working in Mekelle city, Tigray, Ethiopia, 2018. This study is based on an institutional-based cross-sectional study design, where 328 bankers are selected randomly from bankers working in Mekelle city from February to June 2018. Data were entered, organized, and analyzed by SPSS version 23. A final logistic model was run to identify factors associated with WMSDs, and the magnitude and direction of association were decided based on the adjusted odds ratio (AOR) and its corresponding 95% confidence interval (95% CI).

**Result:**

Out of 307 bankers, the annual prevalence rate of WMSDs was 65.5% (201). Significant predictors were being 30–39 years old [AOR = 5.552; 95% CI = 1.465–21.039] and above 40 years old [AOR = 5.719; 95% CI = 1.422–22.994], low educational level [AOR = 4.256; 95% CI = 1.139–15.895], working > 5 years [AOR = 3.892; 95% CI = 1.841–8.231], not doing physical exercises [AOR = 2.866; 95% CI = 1.303–6.304], stress [AOR = 4.723; 95% CI = 2.421–9.213], poor posture [AOR = 2.692; 95% CI = 1.339–5.411], breaks [AOR = 5.170; 95% CI = 2.070–12.912], and ergonomics [AOR = 3.801; 95% CI = 1.260–11.472].

**Conclusion:**

The prevalence of WMSDs among bankers was high. The significant associated factors responsible for the occurrence of work-related musculoskeletal disorders include longer working experience, being above 30 years old, low educational status, physical exercise, job stress, poor posture, absence of breaks during working hours, and absence of ergonomic training.

## 1. Background

Work-related musculoskeletal disorders are an important public health problem in the working environment which may affect one or more of the following: neck, shoulder, elbow, wrist, hand, upper back, low back, hip, knee, ankle, and foot [1–5]. Specifically, low back pain accounted for nearly half all MSDs, and neck pain, a fifth. The proportions of MSD are higher in the aging group compared to the younger group and common in high-income countries than in low-income countries [6, 7]. Musculoskeletal disorders affect all persons regardless of age and sex and are prevalent across a wide range of industries and jobs [8–10].

Literature has shown that musculoskeletal disorders are an important public health problem that accounts between 42% and 58% of all work-related disorders and is the most frequent cause of all health-related absences from work [2]. Abdelu 2012 et al. reported that WMSDs among bank office workers are both quite common and quite disabling with 42% of disability rate [2]. Moreover, a study done in Iran, Bangladesh, Punjab India, and Tamil Nadu reported that the annual prevalence rate of WMSDs among bankers was 78.5%, 69.3%, 83.5%, and 33.8%, respectively [11–14]. A study also done in Kuwait reported the prevalence of work-related musculoskeletal disorders among bankers in the previous week and during the previous year of the study was 57% and 80%, respectively [1].

Working conditions are often presumed to play an important part in an etiology of WMSDs [15]. It is associated with working postures which included bent, static, or maintained postures, twisted posture for long periods, and making repetitive movements with the trunk. Apart from the working conditions, a wide range of risk factors such as age, gender, lifestyle factors such as smoking, psychological stress, alcohol consumption, previous pain symptoms, psychosocial factors, socioeconomic variables, poor muscle flexibility and strength, physical activity, and physical workload have been associated with the development and persistence of different WMSDs [3, 4, 9, 14, 16–23].

Work-related musculoskeletal disorders significantly affect the quality of life and result in losing work time or working absenteeism, increasing work restriction, changing to another job, with considerable compensation, and losses on the individual, the organization, the family and the society levels [10, 24, 25]. Many individual workers appear to have suffered serious economic consequences from WMSDs including losing homes, incurring large out-of-pocket expenses, being divorced, loss of health insurance, and facing economic insecurity due to financial hardship and also a negative social impact on the individual [23, 25]. Studies reported industries with higher prevalence of WMSDS are affected more in terms of lost productivity due to the employee's days away from work because of MSDS. In the case of MSDs causing permanent disabilities, new hiring and training costs are also a part of the losses experienced by the employers [23, 26].

Studies conducted in the past decade show that the prevalence, etiology, and risk factors of WMSDs vary among individuals in different professions, environment, and geographical location [27]. In the study setting, it is presumed that there is a high burden of WMSDs among bankers. However, the exact prevalence and associated factors are not well known among bankers in Ethiopia, particularly in Mekelle. The study helps to identify the risk factors of WMSDs among bankers which are harmful for the bankers. So, the information provided by this study can be used to stress the need for primary prevention of WMSDs, thereby promoting health in the banking industry and economic development of the country. Therefore, this study aims to determine the prevalence and associated factors of WMSDs among bankers working in Mekelle city.

## 2. Methods

### 2.1. Study Design, Period, and Study Area

An institutional-based cross-sectional study design was conducted from February to June 2018 in Mekelle city. Mekelle city is the capital city of Tigray region, which is found about 778 kilometers to the north of Addis Ababa, the capital city of Ethiopia, at a latitude and longitude of 13029′N:39028′E, with an elevation of 2,084 meters above sea level [28]. The city has 98 private and government bank branches with a total of 1320 bankers working in all private and government banks.

### 2.2. Study Population and Sampling Procedure

In this study, bankers are defined as any technical and skilled staff that are involved in the use of computers or other means for data collection, processing, and programming of day-to-day financial and business transactions in the banking sector [3]. All bankers working in all private and governmental banks were eligible for the study except those bankers with any history of injury/trauma, history of surgery in the previous six months, and women with known pregnancy before conducting the current study.

First, banks in Mekelle city were stratified into governmental and nongovernmental banks. Five hundred fifty-eight bankers of governmental banks or commercial banks of Ethiopia were found in 20 branches and a total of 762 bankers of nongovernmental banks were found in 78 branches in Mekelle city with a total of 1320 bankers. A list of bankers was obtained from the district bureau and managers of each bank. Sampling frame was prepared for 558 bankers of governmental and 762 bankers of private banks after excluding those who were not eligible for the study. Then, the sample size was proportionally allocated to the number of bankers in the governmental and nongovernmental banks. Finally, a simple random sampling technique of the lottery method was applied to select the study participants from the sampling frame.

### 2.3. Sample Size Determination

The sample size was determined by the Epi-info version 7 software calculator considering a single population proportion. To determine the minimum sample size for this study, the following assumptions were considered, since there was no study conducted on the prevalence and associated factors of work-related musculoskeletal disorders among bank workers in Ethiopia, though we considered the anticipated prevalence of WMSDs among bankers in Mekelle city = 50%:

 
*n* = minimum sample size,
 
*P* = anticipated prevalence of WMSDs among bankers in Mekelle city = 50%,
 
*d* = margin of error = 5%,
 
*Z* = confidence interval at 95% = 1.96,
 
*P* = number of bankers in Mekelle city = 1320,
 
*n* = 298; so, by adding a 10% nonresponse rate, the final sample size was 328.

### 2.4. Data Collection Tools, Procedure, and Data Quality Management

Data was collected from bankers by interviewing the study participants and was adopted and modified to suit the purpose of the study and environment from the standardized Nordic Musculoskeletal Questionnaire (NMQ) [29] and by revision of different literatures [1–3, 13].

The questionnaire comprised five sections: the first section of the questionnaire was designed to elicit questions on sociodemographic variables. The second section contains questions on individual factors. The third section was about ergonomics and working environment-related factors. The fourth section on the prevalence of WMSDs constituted a picture of human form with nine body areas shaded and defined as follows: neck, shoulders, upper back, lower back, elbows, wrists/hands, thighs, knees, and ankles. The fifth section was about job satisfaction and job stress.

The principal investigator selected five physiotherapists with a BSc degree as data collectors and three physiotherapists with a master's degree as supervisors. The data collectors and supervisors were trained for two days, before they started, on the questionnaire administration, data collection, supervision procedures, informed consent, how to approach bankers, ethical procedures, and objectives of the study.

The quality of data was controlled starting from the time of questionnaire preparation. The questionnaire was first prepared in English and then translated into Tigrigna language and then back to the English version to ensure consistency by a physiotherapist who has good communication in both English and Tigrigna. Training was given for the data collectors and supervisors for two days on how to approach study subjects and use the questionnaire for interviews before the study. After completing the training, a pretest of the questionnaire was done in a 5% sample size out of the study area in Wukro city with a similar population. Finally, a discussion was made about problems encountered during the pretest and it was decided that the questionnaire be modified to ensure the consistency of questionnaires and that all the data collectors had the same understanding. This also helps to conduct good quality study, by gathering data that are reliable, valid, and informative. During data collection, questionnaires were received by supervisors and principal investigators from data collectors and checked for completeness, accuracy, and consistency.

### 2.5. Ethical Approval and Consent to Participate

Ethical approval of the study was obtained from the ethical review committee of Mekelle University, college of health sciences. A formal letter for cooperation was obtained from the Mekelle district bank bureau and managers of respective banks and was included in the study after a necessary clarification about the purpose of the study. Written informed consent was obtained from the study participants after being informed in detail about the objective, purpose, benefits, and risks of the study. Appropriate measures were taken to assure the confidentiality of information both during and after data collection.

### 2.6. Operational definition

#### 2.6.1. Work-Related Musculoskeletal Disorders

In this study, bankers are diagnosed as having WMSDs if there is any pain, ache, or discomfort at any part of their structures in the neck, shoulder, elbow, wrist, hand, upper back, low back, hip, knee, and foot that results from a work-related event for at least one day in the previous one year which is relieved by rest and aggravated by work.

### 2.7. Data Processing and Analysis

The collected data were coded, entered, organized, and analyzed by a Statistical Package for social sciences (SPSS) Version 23.0. Frequency tables or percentages or bar graphs were used to present results of categorical variables and mean (±std.) to summarize continuous variables. Bivariate binary logistic regression analysis was done and thus variables with *p*-value less than 0.25 were considered as potential candidates in the final multivariable logistic regression analysis. Moreover, chi-squared assumptions were checked. Then, a multivariable logistic regression analysis was run to identify associated factors of WMSDs among bankers. Finally, variables with *p* < 0.05 in the final logistic model were considered statistically significant and the strength and direction of association were measured by adjusted odds ratio (AOR) with corresponding 95% confidence interval.

## 3. Result

### 3.1. Sociodemographic Characteristics of Bankers

Three hundred thirty-eight bankers were selected for the study yielding a response rate of 307 (93.6%). The nonresponse was due to lack of time as explained by nonparticipants. From the total of 307 bankers participating in the study, the majority of the participants were male 198 (64.5%) and in the age range of 20–29 years (201 (65.9%)). The mean age of the participants was 29 ± 5 years old and most participants were single (175 (57%)). Regarding the job design, most of them were tellers (210 (68.4%)) and about 216 (70.4%) had less than or equal to five years of working experience. Income statuses of the participants were mean of 7990.28 ± 3092 birr and the majority of them were paid between 5,000 and 10,000 birr (220 (71.7%)). Most participants were holders of a bachelor's degree (243 (79.2%)), and regarding the religious status, the majority (300 (97.7%)) were Orthodox (Table 1).

### 3.2. Individual, Psychosocial, and Lifestyle Factors of WMSDs

Regarding the lifestyle of the bankers, the majority (304 (99%)) were nonsmokers and 263 (85.7%) were nonalcoholic. Regarding the physical activity, the majority (227 (73.9%)) were not doing physical exercise and most of them had a normal BMI (245 (79.8%)). Regarding the psychosocial status, the majority (190 (61.9%)) had no job stress and most of them were satisfied in their job (166 (54.1%)) (Table 2).

### 3.3. Ergonomics and Working Environment-Related Factors of WMSDs

The majority of the bankers (240 (78.2%)) were working in an awkward posture and most of them used adjustable sitting chairs (264 (86%)). Regarding breaks, during working times, most of them had no breaks (266 (86.6%)) and 88 (28.6%) sat in the same position with no change. The majority (283 (92.5%)) had not taken an ergonomic training about postures in the workplace. The median number of customers served each day was 106 persons with a range of 10–500, and most of the participants (125 (40.7%)) were serving between 50 and 100 customers each day (Table 3).

### 3.4. Prevalence of Work-Related Musculoskeletal Disorders among Bankers

**Figure 1:**
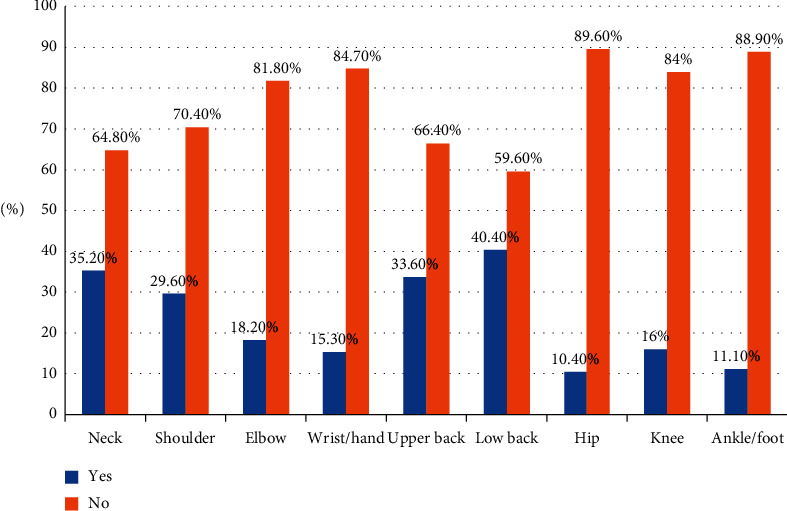
Prevalence of WMSDs distribution in body parts among bankers working in Mekelle city, Tigray, Ethiopia, May 2018 (*n* = 307).

Out of 307 bankers, the annual prevalence rate of WMSDs at any body part was 201 (65.5%), while 108 (35.2%) suffered such disorders during the previous one week.  Figure 1 shows the prevalence of WMSDs by affected body parts among bankers. The current study revealed that the prevalence rate during the 12 months was highest in the lower back (40.4%), followed by neck (35.2%) and the upper back (33.6%) but least in the ankle (11.1%) and hip (10.4%). Male respondents (130 (65.7%)) were reported suffering more from WMSDs than their female counterparts. The prevalence of WMSDs were higher among participants with a single marital status (112 (64%)), in the age range of 20–29 years (133 (66.2%)), and working for ≤ 5 years (125 (57.9%)), and it was higher among tellers (136 (64.8%)) who were paid 5,000–10,000 birr (139 (63.2%)) and among participants with a bachelor's degree (158 (65%)). However, the prevalence of WMSDs was lower in participants who had a good posture during working hours (35 (52.2%)), had breaks (13 (31.7%)), took an ergonomic training (7 (30.4%)), and served a low number of customers each day (42 (67.7%)).

### 3.5. Factors Associated with WMSDs among Bankers

In the bivariate logistic regression analysis, WMSDs were independently associated with age group, educational level, BMI, job stress, job satisfaction, physical exercise, posture, having breaks, and ergonomic training at *p* < 0.25. However, upon fitting the factors in multivariable logistic regression analysis, being 30–39 years old [AOR = 5.552; 95% CI = 1.465–21.039] and above 40 years old [AOR = 5.719; 95% CI = 1.422–22.994], having a low educational level [AOR = 4.256; 95% CI = 1.139–15.895], working > 5 years [AOR = 3.892; 95% CI = 1.841–8.231], not doing physical exercises [AOR = 2.866; 95% CI = 1.303–6.304], stress [AOR = 4.723; 95% CI = 2.421–9.213], poor posture [AOR = 2.692; 95% CI = 1.339–5.411], having breaks [AOR = 5.170; 95% CI = 2.070–12.912], and ergonomics [AOR = 3.801; 95% CI = 1.260–11.472] were independently and significantly associated with WMSDs at *p* < 0.05 in the reduced model (Table 4).

## 4. Discussion

This study determined the prevalence of WMSDs and associated factors among bankers working in Mekelle city, Tigray, Ethiopia. The annual prevalence rate of WMSDs in any body part region was 65.5% while that of the previous one-week prevalence rate was 35.2%. This study was comparable with a study done in Dhaka city, Bangladesh (69.3%) [12]. The possible reason for similarities may be due to a similar sample size (300) and the same cross-sectional study design but the current study's annual prevalence rate was higher than previously reported study in Kancheepuram district, Tamil Nadu, India (33.8%) [10]. The possible reason may be attributed to sociocultural factors, lesser job stress, or understated feedback.

However, this study finding was lower than the annual prevalence rate of WMSDs from studies done in Iran; Punjab, India; Kuwait; Kumasi, Ghana; Maiduguri, Nigeria (78.5%, 83.5%, 80%, 83.5%, and 71.68%, resp.) [3–5, 18, 19]. The prevalence rate of WMSDs from this study during the last seven days was also lower than study findings from Kuwait and Nigeria (57% and 57.52%, resp.) [4, 5]. This lower prevalence might be due to sociocultural factors, lesser job stress, different sampling technique, study design, sample size, definition of WMSDs, study area, workload, and difference in assessment tools of the studies. Additionally, some participants might have underrated their injuries to avoid being labeled “sick” or perceived negatively at their place of work which may affect their promotion or other employment opportunities. A study done in Iran used case study design [19] like in a study done in Nigeria which used a survey study design and participants were recruited by nonprobability sample of convenience from various banks [5] while studies in Kuwait and Kumasi, Ghana, had a relatively higher sample size (800 and 400, resp.) [3, 4], which may lead to a high prevalence rate of WMSDs compared with this study's finding.

Our study revealed that lower back (40.4%), neck (35.2%), upper back (33.6%) and shoulder (29.6%) were the most commonly affected body parts during the previous year. The pattern of distribution of WMSDs in different body parts was similar to other studies which showed that low back, neck, upper back, and shoulder were the most frequent body region reported by bankers [11].

In the present study, it was found that age group was a significant predictor of WMSDs among bankers where respondents who were between 30 and 39 years old were 5.6 times more likely to have WMSDs and those who were above 40 years old were 5.7 times more likely to have WMSDs compared to those aged 20 to 29 years. This study was supported by a study done in Bangladesh [12] where it was shown that increase in age will increase the odds of WMSDs and the possible reason may be an increase in age will increase working experience which, in turn, increases fatigue and muscle tension for many years, finally causing WMSDs. However, the age group was not significantly associated with WMSDs in a study done in Nigeria [3]. The possible reason may be due to a relatively small sample size (226) and a survey study design used.

In this study, the educational status was also significantly associated with developing WMSDS, and those who had a low educational status were 4.2 times more likely to develop WMSDs than those who had a master's degree. The possible reason may be that those who had a low educational status may have less knowledge and fewer skills in terms of ergonomics in the workplace. However, educational status was not significantly associated with WMSDs in other studies of Kuwait, India [1, 12]. The possible reason may be due to differences in the study area and sociocultural difference.

In the current study, psychological stress was positively associated with WMSDs, and WMSDs were higher in those who experienced psychological stress compared with those who never experienced stress at work. Similar studies in Iran also reported that the odds of incidence of WMSDs increase in those who experienced stress [19], which may be due to the cross-sectional study design used. This is due to the fact that high mental and psychological stress may increase muscle tension and decrease micropauses in muscle activity. This may lead to muscle fatigue, even in cases of low loads due to continuous firing of low threshold motor units, which are triggered not only by low-level physical loading but also by mental loading. However, stress was not significantly associated with WMSDs in Nigeria [3]; the possible season may be due to a relatively small sample size and the difference in working environment in a study done in Nigeria [26].

Physical exercise was another factor significantly associated with WMSDs where WMSDs were 2.9 times more likely to occur among participants who were not doing exercise compared to those who exercised. The current study was supported by studies done in Kumasi, Ghana [4], and this may be due to promoting exercise and physical activity and maintaining health and fitness levels and preventing injury.

In this study, breaks between working hours were significantly associated with WMSDs and this is supported by a study done in Punjab, India, and Kumasi, Ghana [13] which imply that respondents who had breaks during working hours have high chances of relaxing their muscles and reducing risks of pain, but breaks were not significantly associated with a study done in Iran [11]. The possible reason may be due to the relatively small sample size (200) used by the study [11].

Furthermore, ergonomic training was a significant factor independently associated with WMSDs among bankers. This study was supported by a similar study done in Kumasi, Ghana [4], and the possible reason may be due to using a similar study design. Besides, poor posture may lead to stiffness and compression in many body regions causing WMSDs.

### 4.1. Strength and Limitations of the Study

This study has significant public health relevance. Hence, the data inform intervention strategies to reduce associated factors for WMSDs. Also, the interview method of data collection was applied in this study. Despite this strength, the main limitation of the study was self-report and participants may not have recalled all incidents of WMSDs. Further, participants may have underestimated their WMSDs to avoid being stereotyped by their superiors or being viewed negatively based on their history of WMSDs, which may affect promotion or future of employment opportunities. Another limitation of the study was that it was conducted using a small sample size.

## 5. Conclusions

The prevalence of WMSDs among bankers was moderate. The significant associated factors responsible for the occurrence of work-related musculoskeletal disorders include longer working experience, being above 30 years old, having a low educational status, lack of physical exercise, job stress, poor posture, absence of breaks during working hours, and not taking ergonomic training.

It is recommended that bankers emphasize comfortable body posture when performing their tasks, do physical exercise, take ergonomic training, and have breaks during working hours as a means of preventive strategies to avoid WMSDs. Besides, further studies with objective measurement, large sample size, and longitudinal study designs are recommended to identify the additional associated factors of WMSDs.

## Figures and Tables

**Table 1:** Sociodemographic characteristics of bankers working in Mekelle city, Tigray, Ethiopia, 2018 (*n* = 307).

Variables	Frequency	Percent (%)	Annual prevalence of WMSDs			
			No		Yes	
			Frequency	Percent (%)	Frequency	Percent (%)
*Gender*						
Male	**198**	**64.5**	**68**	**34.3**	**130**	**65.7**
Female	**109**	**35.5**	**38**	**34.9**	**71**	**65.1**

*Age group*						
20–29	**201**	**65.9**	**68**	**33.8**	**133**	**66.2**
30–39	**91**	**29.8**	**31**	**34.1**	**60**	**65.9**
40 and above	**13**	**4.3**	**7**	**53.8**	**6**	**46.2**

*Working experience (yrs.)*						
**≤5**	**216**	**70.4**	**91**	**42.1**	**125**	**57.9**
**>5**	**91**	**29.6**	**15**	**16.5**	**76**	**83.5**

*Marital status*						
Single	**175**	**57**	**63**	**36**	**112**	**64**
Married	**132**	**43**	**43**	**32.6**	**89**	**67.4**

*Job designation*						
Manager	**10**	**3.3**	**2**	**20**	**8**	**80**
Assistant manager	**24**	**7.8**	**10**	**41.7**	**14**	**58.3**
Auditor	**46**	**15**	**13**	**28.3**	**33**	**71.7**
Casher	**17**	**5.5**	**7**	**41.2**	**10**	**58.8**
Teller	**210**	**68.4**	**74**	**35.2**	**136**	**64.8**

*Level of education*						
Diploma	**30**	**9.8**	**7**	**23.3**	**23**	**76.7**
Bachelor's degree	**243**	**79.2**	**85**	**35**	**158**	**65**
Master's degree	**34**	**11.1**	**14**	**41.2**	**20**	**58.8**

*Religion*						
Orthodox	**300**	**97.7**	**103**	**34.3**	**197**	**65.7**
Others	**7**	**2.3**	**3**	**42.9**	**4**	**57.1**

*Monthly income (birr)*						
<5000	**47**	**15.3**	**12**	**25.5**	**35**	**74.5**
5000–10000	**220**	**71.7**	**81**	**36.8**	**139**	**63.2**
>10000	**40**	**13**	**13**	**32.5**	**27**	**7.5**

**Table 2:** Individual, psychosocial, and lifestyle factors of bankers working in Mekelle city, Tigray, Ethiopia, May 2018 (*n* = 307).

Variables	Frequency	Percent (%)	Annual prevalence of WMSDs			
			No		Yes	
			Frequency	Percent (%)	Frequency	Percent (%)
*Gender*						
Male	**198**	**64.5**	**68**	**34.3**	**130**	**65.7**
Female	**109**	**35.5**	**38**	**34.9**	**71**	**65.1**

*Age group*						
20–29	**201**	**65.9**	**68**	**33.8**	**133**	**66.2**
30–39	**91**	**29.8**	**31**	**34.1**	**60**	**65.9**
40 and above	**13**	**4.3**	**7**	**53.8**	**6**	**46.2**

*Working experience (yrs.)*						
≤5	**216**	**70.4**	**91**	**42.1**	**125**	**57.9**
>5	**91**	**29.6**	**15**	**16.5**	**76**	**83.5**

*Marital status*						
Single	**175**	**57**	**63**	**36**	**112**	**64**
Married	**132**	**43**	**43**	**32.6**	**89**	**67.4**

*Job designation*						
Manager	**10**	**3.3**	**2**	**20**	**8**	**80**
Assistant manager	**24**	**7.8**	**10**	**41.7**	**14**	**58.3**
Auditor	**46**	**15**	**13**	**28.3**	**33**	**71.7**
Casher	**17**	**5.5**	**7**	**41.2**	**10**	**58.8**
Teller	**210**	**68.4**	**74**	**35.2**	**136**	**64.8**

*Level of education*						
Diploma	**30**	**9.8**	**7**	**23.3**	**23**	**76.7**
Bachelor's degree	**243**	**79.2**	**85**	**35**	**158**	**65**
Master's degree	**34**	**11.1**	**14**	**41.2**	**20**	**58.8**

*Religion*						
Orthodox	**300**	**97.7**	**103**	**34.3**	**197**	**65.7**
Others	**7**	**2.3**	**3**	**42.9**	**4**	**57.1**

*Monthly income (birr)*						
<5000	**47**	**15.3**	**12**	**25.5**	**35**	**74.5**
5000–10000	**220**	**71.7**	**81**	**36.8**	**139**	**63.2**
>10000	**40**	**13**	**13**	**32.5**	**27**	**7.5**

**Table 3:** Ergonomics and working environment characteristics of bankers working in Mekelle city, Tigray, Ethiopia, May 2018 (*n* = 307).

Variables	Frequency	Percent (%)	Annual prevalence of WMSDs			
			No		Yes	
			Frequency	Percent (%)	Frequency	Percent (%)
*Posture*						
Good	**67**	**21.8**	**32**	**47.8**	**35**	**52.2**
Poor	**240**	**78.2**	**74**	**30.8**	**166**	**69.2**

*Type of chair*						
Adjustable	**264**	**86**	**91**	**34.5**	**173**	**65.5**
Nonadjustable	**43**	**14**	**15**	**34.9**	**28**	**65.1**

*Break*						
No	**266**	**86.6**	**78**	**29.3**	**188**	**70.7**
Yes	**41**	**13.4**	**28**	**68.3**	**13**	**31.7**

*Ergonomics training*						
No	**283**	**92.5**	**90**	**31.7**	**194**	**68.3**
Yes	**23**	**7.5**	**16**	**69.6**	**7**	**30.4**

*Duration of sitting in same position (min)*						
**<15**	**72**	**23.5**	**25**	**34.7**	**47**	**65.3**
**15–30**	**88**	**28.6**	**40**	**45.5**	**48**	**55.5**
**>30**	**147**	**47.9**	**41**	**27.9**	**106**	**72.1**

*Number of customers*						
**<50**	**62**	**20.2**	**20**	**32.3**	**42**	**67.7**
**50–100**	**125**	**40.7**	**55**	**44.0**	**70**	**56.0**
**>100**	**120**	**39.1**	**31**	**25.8**	**89**	**74.2**

**Table 4:** Bivariate and multivariate logistic regression analysis of associated factors with WMSDs among bankers working in Mekelle city, Tigray, Ethiopia, May 2018 (*n* = 307).

Variables	WMSDs		Bivariate	Multivariate		
	No	Yes	Corollary (95% CI)	*P*-value	AOR (95% CI)	*P*-value
*Age group*						
20–29	68 (33.8%)	133 (66.2%)	1	1		
30–39	31 (34.1%)	60 (65.9%)	2.282 (0.738–7.056)^*∗*^	0.152	**5.552 (1.465–21.039)** ^*∗∗*^	**0.012**
40 and above	7 (53.8%)	6 (46.2%)	2.258 (0.698–7.301)^*∗*^	0.174	**5.719 (1.422–22.994)** ^*∗∗*^	**0.014**

*Educational level*						
Diploma	7 (23.3%)	23 (76.7%)	2.3 (0.775–6.823)^*∗*^	0.133	**4.256 (1.139–15.895)** ^*∗∗*^	**0.031**
Bachelor's degree	85 (35%)	158 (65%)	1.301 (0.626–2.706)	.481	2.023 (0.804–5.089)	0.134
Master's degree	14 (41.2%)	20 (58.8%)	1	1	1	1

*Working experience*						
<5	91 (42.1%)	125 (57.9%)	1	1	1	1
5 and above	15 (16.5%)	76 (83.5%)	3.689 (1.992–6.830)^*∗*^	<0.001	**3.892 (1.841–8.231)** ^*∗∗*^	**<0.001**

*BMI*						
Underweight	16 (53.3%)	14 (46.7%)	1	1		
Normal	82 (33.5%)	163 (66.5%)	0.292 (0.100–.854)^*∗*^	.025		
Overweight	8 (25.0%)	24 (75.0%)	0.663 (0.285–1.539)	.339		

*Physical exercise*						
No	78 (29.8%)	184 (70.2%)	3.885 (2.012–7.504^*∗*^	<0.001	**2.866 (1.303–6.304)** ^*∗∗*^	**0.009**
Yes	28 (62.2%)	17 (37.8%)	1	1	**1**	

*Job stress*						
No	86 (45.3%)	104 (54.7%)	1	1		
Yes	20 (17.1%)	97 (82.9%)	4.011 (2.292–7.019)^*∗*^	<0.001	**4.723 (2.421–9.213)** ^*∗∗*^	**<0.001**

*Job satisfaction*						
No	42 (29.8)	99 (70.2)	1.479 (0.918–2.384)^*∗*^	0.108		
Yes	64 (38.6)	102 (61.4)	1	1		

*Posture*						
Bad	74 (30.8%)	166 (69.2%)	2.051 (1.181–3.562)^*∗*^	0.011	**2.692 (1.339–5.411)** ^*∗∗*^	**0.005**
Good	32 (47.8%)	35 (52.2%)	1	1		

*Break during working hours*						
No	78 (29.3%)	188 (70.7%)	5.191 (2.555–10.546)^*∗*^	<0.001	**5.170 (2.070–12.912)** ^*∗∗*^	**<0.001**
Yes	28 (68.3%)	13 (31.7%)	1	1	**1**	**1**

*Ergonomic training*						
No	90 (31.7%)	194 (68.3%)	4.927 (1.958–12.397)^*∗*^	0.001	**3.801 (1.260–11.472)** ^*∗∗*^	**0.018**
Yes	16 (69.6%)	7 (30.4%)	1	1		

## Data Availability

The data used in this study are available upon reasonable request from the corresponding author.
